# Experimental characterization of Raman overlaps between mode-groups

**DOI:** 10.1038/srep34693

**Published:** 2016-10-05

**Authors:** Erik N. Christensen, Jacob G. Koefoed, Søren M. M. Friis, Mario A. Usuga Castaneda, Karsten Rottwitt

**Affiliations:** 1 Department of Photonics Engineering, Technical University of Denmark, 2800 Kongens Lyngby, Denmark

## Abstract

Mode-division multiplexing has the potential to further increase data transmission capacity through optical fibers. In addition, distributed Raman amplification is a promising candidate for multi-mode signal amplification due to its desirable noise properties and the possibility of mode-equalized gain. In this paper, we present an experimental characterization of the intermodal Raman intensity overlaps of a few-mode fiber using backward-pumped Raman amplification. By varying the input pump power and the degree of higher order mode-excitation for the pump and the signal in a 10 km long two-mode fiber, we are able to characterize all intermodal Raman intensity overlaps. Using these results, we perform a Raman amplification measurement and demonstrate a mode-differential gain of only 0.25 dB per 10 dB overall gain. This is, to the best of our knowledge, the lowest mode differential gain achieved for amplification of mode division multiplexed signals in a single fiber.

During the past decade, the increase in data capacity per fiber has slowed relative to the rapid progress in the 1990’s while, at the same time, the demand for capacity continues to grow exponentially^1^. Current methods for signal multiplexing, i.e. wavelength-, polarization-, time-, and quadrature-division multiplexing, are approaching their fundamental limits so new means of multiplexing are needed. Space-division multiplexing^2^ in the form of multi-core fibers has already been used to achieve new heights in data capacity from a single laser source^3^,^4^; in single-core fibers supporting multiple spatial modes, long-distance propagation of optical signals has been demonstrated^5^,^6^; and recently, data transmission in a few-mode multi-core fiber was presented^7^. One important challenge of mode-division multiplexing (MDM) systems is building multi-mode optical amplifiers, that have mode-equalized amplification of all spatial modes, to compensate for example for distributed fiber loss; it is desirable for a multi-mode amplifier to avoid mode-dependent gain (MDG) in order to maximize capacity^8^. As for traditional single-mode systems, discrete Erbium-doped fiber amplifiers have been applied to multi-mode systems as well and low MDG has been achieved for some of the modes in fibers with specially designed Erbium-doping profiles^9^,^10^,^11^.

Another approach to counter-balance fiber losses is distributed Raman amplification, which is also widely used already in single-mode networks due to its superior noise properties in the backward-pumped configuration^12^. Furthermore, it has been shown theoretically that minimal MDG is possible by coupling pump power into a specific combination of spatial modes^13^, or by optimizing fiber design^14^, which makes Raman amplifiers a promising candidate for realizing low-loss, multi-mode transmission links over large distances.

Earlier work has demonstrated Raman gain between higher-order modes with the pump in only one mode^5^,^15^,^16^. Besides the obvious challenges related to exciting the pump in a specific combination of modes, it may often also prove difficult to determine the exact mode combination that leads to the lowest possible MDG because the required fiber data are unavailable from the fiber supplier. In this paper, we present an experimental characterization of the intermodal Raman intensity overlap of the guided modes of a two-moded (6 modes counting polarisation and LP_11a_ and LP_11b_) few-mode fiber (FMF) using mechanically induced long-period gratings (LPGs) to excite the higher-order modes. Using the obtained results, we demonstrate backward pumped Raman amplification of a continuous wave (CW) signal through 10 km of a two-moded fiber with a very low MDG of 0.25 dB per 10 dB gain by pumping in a combination of the LP_01_ and LP_11_ modes. The mode-differential gain obtained required no prior knowledge about the Raman intensity overlaps of the fiber.

## Results

The purpose of the present work is to characterize the intermodal Raman overlaps and use them to achieve a minimal MDG in a backward-pumped Raman fiber amplifier. This is done by coupling the pump light into the fiber in the correct combination of the LP_01_- and LP_11_-modes. As will be discussed in the Methods section below, due to strong mode-coupling, the two-fold quasi-degenerate LP_01_-modes and four-fold quasi-degenerate LP_11_-modes are simply considered as two distinct groups of modes. We carry out two measurements: Firstly, the Raman gain of a continuous wave signal in the LP_01_-mode is measured vs. total pump input power for five different modal compositions of the pump, i.e. different combinations of the LP_01_- and the LP_11_-modes. Secondly, both pump and signal are converted to LP_11_. This data is used to calculate the Raman intensity overlaps relative to the LP_01_-LP_01_-overlap, which is all that is needed to find the correct combination of pump modes.

### Raman intensity modal overlaps

**Figure 1:**
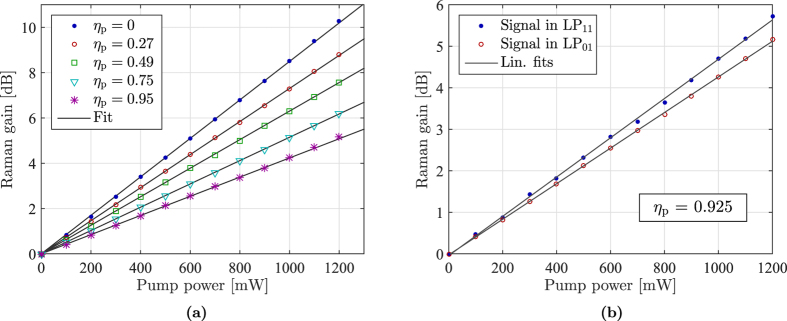
(**a**) Measured Raman gain vs. input pump power for five different pump conversion degrees, *η*_p_; the lines result from the two-parameter fit evaluated at each conversion degree. (**b**) Measurements of Raman gain for signal and pump in LP_11_. For comparison is shown the measurement with pump in LP_11_ and signal in LP_01_.

Assuming both pump, and signal to be CW sources, the signal power 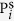, in spatial mode *i*, and the counter propagating 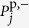 and copropagating pump power 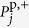, in spatial mode *j*, is governed by^17^


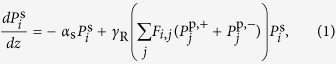



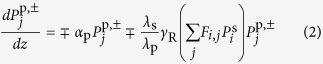


where *α*_s_ and *α*_p_ are loss coefficients for signal and pump wavelengths *λ*_s_ and *λ*_p_, and g_R_ is related to the spontaneous Raman scattering cross section. Note that *γ*_R_ and *α*_p,s_ are assumed mode-independent. The intensity overlap integrals are defined as


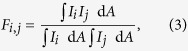


with *I*_*i*_ being the intensity of mode *i* integrated over the entire fiber cross section. Solving (1) and (2) using the undepleted pump approximation, we arrive at an expression for the on/off gain


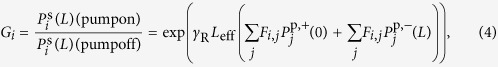


where *L*_eff_ = (1 − exp[−*α*_p_*L*])/*α*_p_ is the effective fiber length and *L* is the physical fiber length. The setup used is a backwards pumped configuration, where the pump has only two different spatial profiles (corresponding to LP_01_ and LP_11_), so Eq. (4) can be reduced to


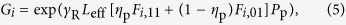


for the signal in mode *i*, where *η*_p_ is the degree of conversion of the pump from LP_01_ to LP_11_ (*η*_p_ = 0 when all the pump power is in LP_01_, and *η*_p_ = 1 when all the pump power is in LP_11_) and *P*_p_ is the total input pump power (in all modes). Using the setup which is described in the methods section below, 65 measurements were carried out with 5 different conversion degrees and 13 different pump power levels varying from 0 to 1200 mW for each conversion degree. From the expected form of the gain, Eq. (5), we fitted a function of the form


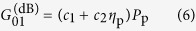


to the data, where *c*_1_ and *c*_2_ are fitting parameters. The result is presented in Fig. 1a where data and fitting lines are shown at the five different values of *η*_p_. The obtained values for the fitting parameters are *c*_1_ = 8.50 d*B*/*W* and *c*_2_ = −4.48 d*B*/*W*. The theoretical expression is in excellent agreement with the obtained data with these values of the fitting parameters. From these values the ratio of the Raman intensity overlaps between the LP_01_-LP_01_-modes and LP_01_-LP_11_-modes is obtained


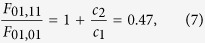


by comparing (5) and (6). This result agrees well with the value of 0.48 obtained from simulated mode-profiles provided by the fiber supplier.

Subsequently, the signal was coupled to the LP_11_-mode with the highest attainable efficiency, (*η*_*p*_ > 0.99), and the pump was converted to the LP_11_-mode with an efficiency of *η*_p_ = 0.925, see the Methods section for details, and the Raman gain of the LP_11_-signal was measured vs. the input pump power. A linear function of the type


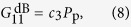


was fitted to the data. From this the ratio 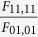 is calculated, taking into account the pump conversion degree. The slope obtained from the fit to the LP_11_-LP_11_ -data was *c*_3_ = 4.74 dB/W. We assume wavelength independence of the overlap integrals (i.e. that the LP_01_-LP_11_ and LP_11_-LP_01_ overlaps are nearly identical). By comparison of Eqs (8) and (6) to (5) we note that *c*_1_ = *kF*_01,01_ and *c*_3_ = *k*(*F*_01,11_ + *η*_p_(*F*_11,11_ − *F*_01,11_)) with 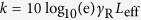. Using Eq. (7) for the ratio *F*_01,11_/*F*_01,01_, these two expressions can be rearranged to give


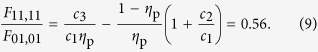


This is compared to the simulated values for LP_11a_-LP_11a_ and LP_11a_-LP_11b_ of 0.72 and 0.24, respectively. The measured overlap is, as expected, an intermediate value that depends on the mode-coupling within the LP_11_ mode-group. In Table 1 the measured overlaps are summarized, and in Table 2 the simulated overlaps are shown. Notice that the overlaps are normalized so that the LP_01_-LP_01_-overlap equals one.

### Mode-equalized Gain Based on Measured Overlaps

**Figure 2:**
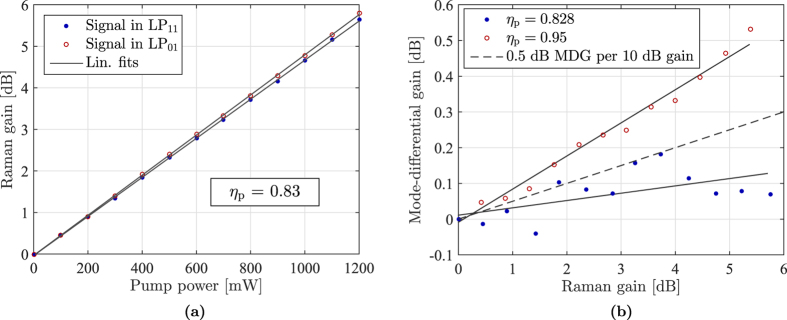
(**a**) Raman gain for both a LP_01_ and LP_11_-signal with a pump in the mixture of 83% LP_11_ and 17% LP_01_ demonstrating near-equal gain for the two signals. (**b**) MDG between the two signal modes for pump almost completely converted to LP_11_ and pump in the mixture of 83% LP_11_ and 17% LP_01_. A line for 0.5 dB gain difference per 10 dB gain is included for reference.

Since the LP_11_-LP_11_ and LP_11_-LP_01_ intensity overlaps often turn out to be very similar in FMFs, relatively low differential gain can be obtained by simply launching the pump completely into LP_11_. This was experimentally verified by R. Ryf *et al*.^6^ where a differential gain of 0.5 dB per 10 dB of gain was observed. For the fiber used in this work, such a scheme results in a differential gain of 1 dB per 10 dB of gain as obtained from the data shown in Fig. 2b (the differential gain in the figure is slightly lower since the pump is only converted 95% into LP_11_). Using our knowledge of the intensity overlap integrals, the condition for equal signal gain across the two signal-modes, *G*_11_ = *G*_01_, can be written as


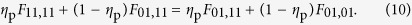


This equation can be solved, using the experimentally obtained values for the ratios *F*_11,11_/*F*_01,01_ and *F*_01,11_/*F*_01,01_, to obtain an equal-gain pump conversion of


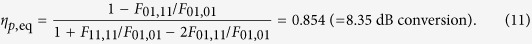


In Fig. 2a the results of measuring a signal launched first completely in LP_01_ and then completely in LP_11_ with a pump conversion of *η*_p_ = 0.83, i.e. slightly below the optimal value, are shown. From the figure it is clear that very little mode-dependent gain remains (compare with Fig. 1b). The mode-differential gain as a function of the mean gain is seen in Fig. 2b, showing a residual MDG of only 0.25 dB per 10 dB of Raman gain as obtained from the fitted lines. This differential mode gain is, to the best of our knowledge, the lowest that has so far been experimentally demonstrated. The reason for the fluctuation in MDG is most likely due to mode coupling between LP_11a_ and LP_11b_. The LPG preferentially couples to the LP_01_ mode that we detect in the optical spectrum analyzer (OSA), as explained in the Methods section. This means that any mode coupling between LP_11a_ and LP_11b_ shows up as a small variation in the measured amplified signal. In the *η*_p_ = 0.83 (blue dot) measurement the back coupling is slightly more unstable compared to the *η*_p_ = 0.95 (red circle). This is due to the different configuration of the back coupling LPG.

## Methods

**Figure 3:**
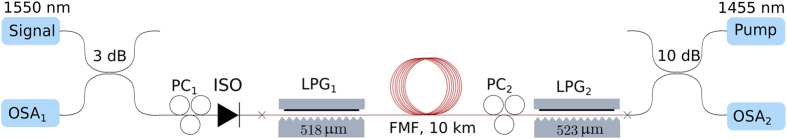
The experimental setup used for all measurements. ISO: Isolator, OSA: optical spectrum analyser, PC: polarization controller, LPG: long period grating, FMF: few-mode fiber. Red lines signify FMF and black lines single mode fiber with crosses indicating splices. The number on the gratings indicate pitch.

The intermodal Raman gain is measured using the experimental setup shown in Fig. 3. The setup is a distributed backwards pumped multi-mode Raman amplifier with a CW laser operated at 1550 nm as the signal source, and an unpolarized 1455 nm Raman fiber laser used for optical pumping. The characterized fiber is a 10 km, 2-moded graded-index fiber.

### Higher-Order Mode excitation

The excitation of higher-order modes is achieved by use of mechanically induced LPGs, which are created by pressing the fiber between a periodically grooved aluminum block and a rubber pad. This creates a periodic perturbation in the fiber index, which induces mode coupling if the pitch of the induced gratings matches the difference in propagation constants of the modes^18^.

Using a broadband supercontinuum source at the signal input the mode-converted wavelengths are observed in the OSA_2_ as a drop in the power spectrum due to the FMF to single mode fiber splice working as a mode filter. The effective pitch of the LPG is changed by adjusting the angle of the grooves with respect to the fiber, until maximum mode-conversion is achieved at the signal wavelength. The use of a supercontinuum source for calibration is not strictly necessary if the difference in propagation constant for the modes of interest is known, but it facilitates the excitation process. Based on the knowledge of the propagation constants the pitch for the pump wavelength was calculated to be 527 μm, which is in excellent agreement with the 523 μm pitch experimentally observed at maximum conversion. The LPGs are polarization dependent^18^, so a polarization controller (PC) is used to optimize conversion of the polarized signal source. After propagation through the fiber the signal is converted back to the fundamental mode using a second LPG.

From standard mode-coupling theory the coupling strength between the modes in a step-index fiber is given by^19^


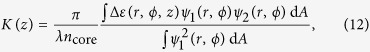


where *ψ*_1,2_ are the scalar mode profiles of the fiber. Since the grooves of the mechanical block are only applied to the fiber from one direction, the perturbation Δ*ε*(*r*, *ϕ*, *z*) is asymmetric with respect to this direction. Since the LP_01_ mode is a circularly symmetric mode, we expect that mainly the LP_11_ mode which is spatially asymmetric with respect to the pertubation direction is excited in the induced grating. However, since we use an unpolarized pump, both polarizations of this spatial mode are excited resulting in an almost equal excitation of the four full-vectorial modes (TE_01_, TM_01_, HE_21*a*_ and HE_21*b*_) that constitute the pseudo-LP_11_ modes. The strong coupling between these modes is expected to quickly smooth out any difference in the excitation^17^. Thus, following a similar approach as Antonelli *et al*.^20^, we only consider the excitation of the quasi-degenerate groups of modes, LP_01_ and LP_11_, consisting of two and four nearly degenerate modes, respectively. In this regard, the measured overlaps are essentially an average over these groups.

### Characterization of fiber under test

For all measurements the signal power launched is 0.4 mW, and the launched pump power is varied from 0 to 1200 mW. For each pump power the on/off gain is measured by OSA_2_. The ratio of the LP_01_-LP_01_ and LP_01_-LP_11_ overlaps is found with the signal in LP_01_ and the pump in varying mixtures of both LP_11_ and LP_01_ by adjusting LPG_2_ to the desired pump mode conversion.

For the LP_11_-LP_11_ gain measurement LPG_1_ and PC_1_ were adjusted to obtain more than 99% signal conversion, and LPG_2_ was adjusted to obtain a maximum of *η*_*p*_ = 0.92 pump conversion; The lower pump conversion is due to the pump being unpolarized. The LPG_2_ conversion bandwidth is large enough such that, by optimizing PC_2_, 12 dB of the signal is converted back to LP_01_. The back conversion is necessary due to the mode-filtering effect of the single-mode to multi-mode fiber splice. The gain of the back converted signal is the LP_11_-LP_11_ gain.

### Equal modal gain measurement

To equalize the modal gain, we first adjust LPG_2_ so that we are pumping in a combination of the LP_11_ and LP_01_ modes very close to the optimal value 85% conversion as obtained from the previous measurements, see Eq. (11). We then first adjust LPG_1_ and PC_1_ to maximize signal conversion (*η*_*p*_ > 0.99) and measure the gain of this mode. Then LPG_1_ is lifted so that the signal is a pure LP_01_-mode and the gain for this mode is measured. The difference in the gain for these two signal-modes then gives the mode-differential gain.

## Conclusion

We have experimentally characterized the intermodal Raman overlaps in a few-mode fiber by varying the launched pump power and the conversion efficiencies of the pump and signal using mechanically induced long-period gratings for mode excitation. The overlap integrals (relative to the LP_01_-LP_01_ overlap) for all modal combinations were obtained in this way for a specific few-mode fiber. By use of the obtained overlaps, it was further demonstrated how a mode-differential gain of only 0.25 dB per 10 dB overall gain is obtained by pumping in a specific combination of the LP_11_ and LP_01_ modes. In the specific few-mode fiber under test, the differential gain was shown to be significantly lower when pumping in the determined combination of modes compared to when pumping only in LP_11_.

## Additional Information

**How to cite this article**: Christensen, E. N. *et al*. Experimental characterization of Raman overlaps between mode-groups. *Sci. Rep*. **6**, 34693; doi: 10.1038/srep34693 (2016).

## Figures and Tables

**Table 1:** Measured values for overlap integrals relative to the LP_01_-LP_01_ overlap.

	LP_01_-LP_01_	LP_11_-LP_01_	LP_11_-LP_11_
Measurement	1	0.47	0.56

**Table 2:** Simulated overlap integrals for all modes.

	LP_01_	LP_11a_	LP_11b_
LP_01_	1	0.48	0.48
LP_11a_	0.48	0.72	0.24
LP_11b_	0.48	0.24	0.72
